# Numerical optimization of microfluidic vortex shedding for genome editing T cells with Cas9

**DOI:** 10.1038/s41598-021-91307-y

**Published:** 2021-06-03

**Authors:** Justin A. Jarrell, Brandon J. Sytsma, Leah H. Wilson, Fong L. Pan, Katherine H. W. J. Lau, Giles T. S. Kirby, Adrian A. Lievano, Ryan S. Pawell

**Affiliations:** 1 Indee Labs, Berkeley, CA USA; 2 Future Industries Institute, University of South Australia, Mawson Lakes, SA Australia

**Keywords:** Transfection, Immunotherapy

## Abstract

Microfluidic vortex shedding (*µVS*) can rapidly deliver mRNA to T cells with high yield and minimal perturbation of the cell state. The mechanistic underpinning of *µVS* intracellular delivery remains undefined and *µVS*-Cas9 genome editing requires further studies. Herein, we evaluated a series of *µVS* devices containing splitter plates to attenuate vortex shedding and understand the contribution of computed force and frequency on efficiency and viability. We then selected a *µVS* design to knockout the expression of the endogenous T cell receptor in primary human T cells via delivery of Cas9 ribonucleoprotein (RNP) with and without brief exposure to an electric field (*eµVS*). *µVS* alone resulted in an equivalent yield of genome-edited T cells relative to electroporation with improved cell quality. A 1.8-fold increase in editing efficiency was demonstrated with *eµVS* with negligible impact on cell viability. Herein, we demonstrate efficient processing of 5 × 10^6^ cells suspend in 100 µl of cGMP OptiMEM in under 5 s, with the capacity of a single device to process between 10^6^ to 10^8^ in 1 to 30 s. Cumulatively, these results demonstrate the rapid and robust utility of *µVS* and *eµVS* for genome editing human primary T cells with Cas9 RNPs.

## Introduction

Intracellular delivery is a critical process in medicine and biology where exogenous materials are delivered across the cell membrane and into the cytosol. The intracellular delivery of constructs (i.e. DNA, RNA, protein and complexes) into cells allows for fundamental and exploratory research in cellular biology along with the synthesis of engineered cells as therapies^1–3^. The development of a simple and scalable microfluidic technology for intracellular delivery has the potential to dramatically improve the discovery process in research while also accelerating the development and manufacture of life-extending T cell immunotherapies like chimeric antigen receptor T cells (CAR-T)^4^ and T cell Receptor T cells (TCR-T)^5^.

*µVS* represents a simple and scalable approach to intracellular delivery^6^. Based on the fluid dynamics phenomena of vortex shedding, *µVS* is induced as fluid flows past a bluff body (i.e., a micron-scale post in a microfluidic device) creating alternating low-pressure regions downstream of the bluff body. The hydrodynamic conditions created by *µVS* are capable of permeabilizing the lipid bilayer of the cellular membrane to induce transient poration and permit intracellular delivery^6^. Despite these promising characteristics and potential, the fundamental physics contributing to *µVS* remains unclear. Thus, the development of *µVS* as an effective platform for cellular modification requires an improved understanding of vortex shedding to apply *µVS* to the intracellular delivery of additional constructs, cell types and indications.

To this end, we developed a series of three-dimensional, transient, single-phase computational fluid dynamics models of *µVS*. We generated *µVS* designs that attenuate vortices through the addition of various ‘splitter plates’ between post columns of a six-column post array^7^. Using our computational fluid dynamics (CFD) model, we demonstrated that reductions in splitter plate lengths correlated with hydrodynamic conditions to enhance vortex shedding. These effects were subsequently verified through the fabrication of splitter plate devices and delivery of eGFP mRNA to activated primary human T cells via *µVS* (see “Methods”). To explore its potential broader applications, we then applied *µVS* to the delivery of Cas9 and sgRNA targeting the T cell receptor alpha locus (TRAC-1), as a Cas9–RNP complex and successfully knocked out the expression of the endogenous T cell receptor of activated primary human T cells.

To better understand the influence of *µVS* on T cell phenotype, a direct comparison to electroporation was performed evaluating delivery efficiency, cell viability, proliferation, and levels of CD25, PD-1, and IFNγ. This demonstrated that *µVS* results in an equivalent yield of genome-edited T cells shortly after intracellular delivery while maintaining the native T cell phenotype relative to electroporation. We also explored the addition of an electric field to *µVS* (*eµVS*) and increased initial editing efficiencies by 1.8-fold with negligible effect on T cell viability over the course of 14 days. 5 × 10^6^ cells were processed in less than 5 s and *µVS* devices could typically process 10^6^ to 10^8^ cells in 1 to 30 s (not shown). Cumulatively, this demonstrates *µVS* and *eµVS* are rapid and robust alternatives to electroporation for genome editing activated human primary T cells.

## Results

### Hydrodynamic simulations demonstrate reductions in vortex shedding with the inclusion of splitter plates

**Figure 1:**
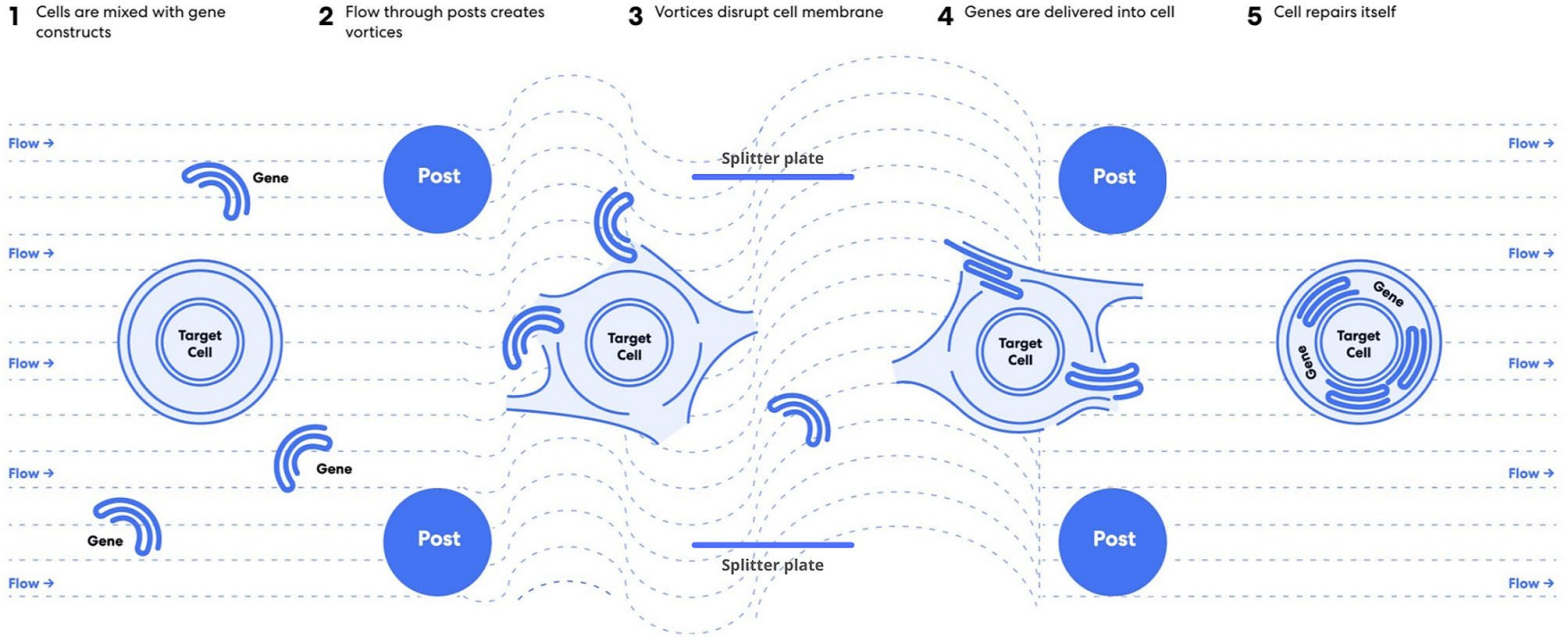
Illustration of the *µVS* intracellular delivery mechanism with a 0.25 splitter plate post array design. Figure generated using Adobe Illustrator Creative Cloud (adobe.com/products/illustrator.html).

**Figure 2:**
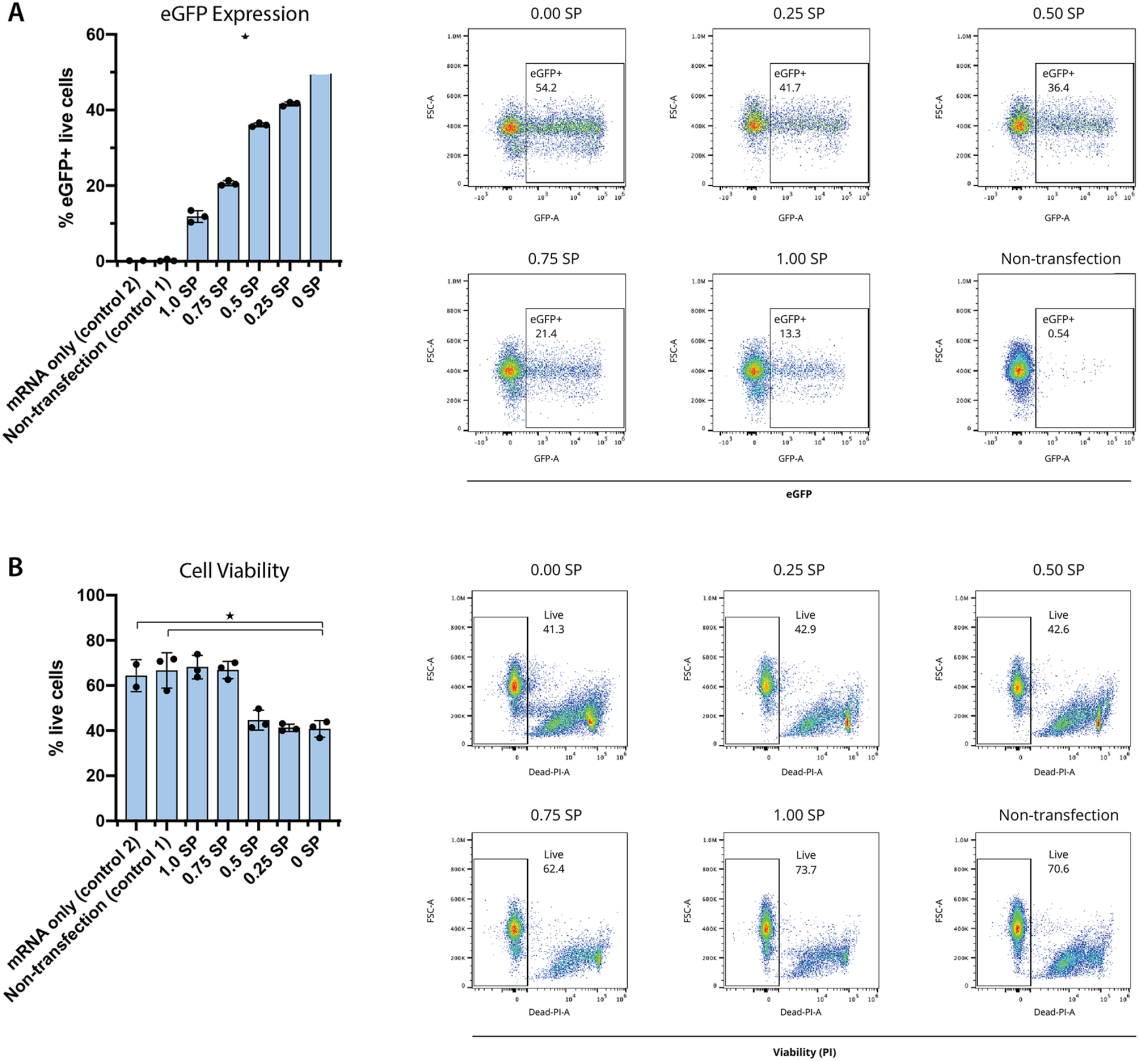
Magnitude of vortex shedding correlates with eGFP expression and cell viability. eGFP-encoding mRNA was delivered to activated primary human CD3^+^ T cells via *µVS* with microfluidic devices containing splitter plates (SP) of various lengths. Splitter plate ratio is defined as the ratio between the length of the splitter plate and the center-to-center distance of two consecutive cylindrical posts in the flow-wise direction. Levels of (**A**) eGFP expression and (**B**) cellular viability were quantified at 24 h post-transfection. Data represent the mean ± standard deviation of n = 3 samples per condition and ≥2 independent experiments. Dot plots representative of triplicate samples with SP ratios and control indicated in each plot. *P < 0.05 by Kruskal–Wallis. Figure generated using Adobe Illustrator Creative Cloud (adobe.com/products/illustrator.html) and GraphPad Prism 9 (graphpad.com/scientific-software/prism/).

Splitter plates of various length were incorporated into a previously reported *µVS* device design^6^ to evaluate the mechanistic underpinning of microfluidic vortex shedding. Empirical measurements of the inlet and outlet pressures and single-phase fluid properties indicated an object Reynolds number of 127 similar to the previously reported experiments^6^. Splitter plate ratio or splitter ratio was defined as the ratio between the length of the splitter plate (i.e. a thin object between two consecutive columns of posts) and the center-to-center distance of two consecutive cylindrical posts in the flow-wise direction. Separation ratio was defined as the ratio of the distance between the center of the cylindrical post (in the 2D-plane, this is the center of the circle) and the leading edge of the splitter plate to the diameter of the cylindrical post^7^. Together, these ratios resolved five device designs, each with a unique splitter plate ratio: 0, 0.25, 0.50, 0.75, and 1.0 (see Figs. 1, 2 and Supplemental Figure 1). The respective separation ratios for these devices are found in Table 1.

**Table 1:** Summary of splitter plate device designs and_,_ simulations.

Device designs			Simulations		
Design	Splitter ratio	Separation ratio	Vortex frequency (kHz)	Spanwise fluctuating hydrodynamics forces (µN)	Post-near wake indicator (PNWI) (%)
SIM01	1.0	0	–	3.9	1
SIM02	0.75	0.75	–	6.4	2
SIM03	0.50	2	–	8.7	3
SIM04	0.25	3.25	36.0	134	40
SIM05	0	10	13.5	336	100

CFD simulation results provided computed flow data including hydrodynamic pressure, flow velocity, and microfluidic vorticity to evaluate vortex strength, structures and shedding behavior of simulated flows within *µVS* devices (Supplemental Method 1). Simulations indicated that both spanwise hydrodynamics fluctuating force and Post Near Wake Indicator (PNWI, Supplemental Method 2) increased with reductions in splitter plate ratios (Table 1, Supplemental Figure 2A,B). In fact, devices with a splitter plate ratio ≥ 0.50 showed minimal to no vortex shedding due to computed vortex attenuation. The total spanwise hydrodynamic fluctuating forces in 1.0, 0.75, 0.50 splitter plate ratio devices was determined to be approximately 3.9 µN, 6.4 µN, and 8.7 µN, respectively. The magnitude of these fluctuations were order(s) of magnitude greater with 0.25 and 0.0 splitter plate ratio devices at approximately 134 µN and 336 µN, respectively. Similarly, the PNWI values of 0.25 and 0.0 splitter plate ratio devices were also determined to be greater than devices with splitter plate ratios ≥0.50 at 40% and 100%, respectively (Table 1). Both increased PNWI and fluctuating force results indicate a substantial increase in vortex ‘strength’ with a decrease in splitter plate lengths. Based on this, we observed the highest vortex ‘strength’ in simulated devices without splitter plates. The measurements also suggested that when splitter plates were placed sufficiently close to the trailing edge of the cylindrical posts, vortex shedding failed to develop^7^. In general, our simulations demonstrated a consistent inverse correlation in PNWI, spanwise hydrodynamics fluctuating forces, and the splitter plate or separation ratio.

### Vortex shedding correlates with delivery efficiency and cell viability

Our CFD simulations highlighted key hydrodynamic features that enhance vortex shedding and could promote intracellular delivery. Therefore, we evaluated these results empirically by fabricating and testing *µVS* devices with splitter plates of various lengths for intracellular delivery of eGFP mRNA to activated primary human CD3^+^ T cells (Figs. 1 and 2). As predicted in our simulations, we observed incremental decreases in eGFP expression with increases in splitter plate lengths, ranging from 51.8 to 11.8% expression between 0 and 1.0 splitter plate ratio devices, respectively (Fig. 2A; Table 2). Conversely, we observed an increase in cell viability with decreases in splitter plate lengths attributed to the attenuation of fluid forces that diminish vortex shedding. However, unlike eGFP expression, the influence of vortex shedding on cell viability was stepwise with the largest change in viability occurring between 0.5 and 0.75 splitter plate ratio devices with 44.6% and 66.9% live cells quantified, respectively (Fig. 2B; Table 2). When compared to our numerical CFD simulations, our experimental results indicate that the presence and strength of computed total spanwise fluctuating hydrodynamic forces is positively correlated with intracellular delivery efficiency with a stepwise decrease in initial cell viability at approximately the onset of vortex shedding. The preferred design with a 0.0 splitter plate was then selected for use in genome editing experiments using Cas9 and *µVS.*

**Table 2:** eGFP expression and cell viability at 24 h post-*µVS* with splitter plate devices.

Device designs			Expression and viability	
Design	Splitter ratio	Separation ratio	Efficiency (%)	Viability (%)
SIM01	1.0	0	11.8 ± 1.55	68.2 ± 5.19
SIM02	0.75	0.75	20.6 ± 0.68	66.9 ± 3.85
SIM03	0.50	2	36.1 ± 0.50	44.6 ± 4.44
SIM04	0.25	3.25	41.6 ± 0.53	41.3 ± 1.62
SIM05	0	10	51.9 ± 2.21	40.8 ± 3.63

### Genome editing with Cas9 and *µVS*

**Figure 3:**
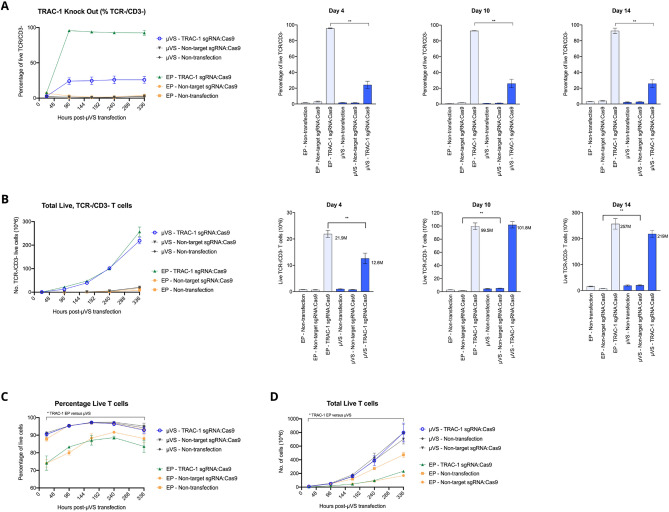
Persistent knockout of endogenous TCR in primary human T cells with Cas9 and *µVS*. Cas9 and locus-specific TRAC-1 gRNAs (as Cas9–RNP complexes) were delivered to activated primary human CD3^+^ T cells via *µVS* or electroporation. Quantification of (**A**) percentage of TCR knockout cells, (**B**) total viable, TCR knockout cells, (**C**) percentage of viable T cells and (**D**) total viable T cells were performed at Days 1, 4, 7, 10 and 14 post-transfection. TCR expression was measured via CD3 and TCRα/β co-staining. TCR knockout was quantified as a percentage of CD3- and TCRα/β-double negative cells and the total number of live cells. Propidium iodide exclusion gating and event collection rate were used to measure viability and cell concentration, respectively. Non-targeting gRNA as a Cas9–RNP complex served as a non-editing control. Non-transfected samples served as a negative control. Data represents mean ± SD of n = 3 samples per condition and ≥ 2 independent experiments. *P < 0.05, **P < 0.01 by unpaired, two-tailed, heteroscedastic *t* tests. Figure generated using Adobe Illustrator Creative Cloud (adobe.com/products/illustrator.html) and GraphPad Prism 9 (graphpad.com/scientific-software/prism/).

To explore the broader application of *µVS* beyond intracellular delivery of eGFP mRNA, we evaluated the utility of *µVS* for Cas9-based T cell engineering relative to electroporation. Using both *μVS* and electroporation, we delivered Cas9 and single-guide RNAs (sgRNAs), as a Cas9–RNP complex targeting the first exon of the TCRα constant region (TRAC-1) to 5.0 × 10^6^ activated primary human CD3^+^ T cells. As a proxy for delivery efficiency, we evaluated TCRα/β and CD3 co-expression in the live cell populations. We quantified co-expression at 4 days after transfection and observed a significant decrease in TCR/CD3 expression with *µVS* and electroporation, with an average editing efficiency of 25% and 95% of live T cells, respectively, compared to cells transfected with a non-target sgRNA-Cas9 RNP and non-transfection controls (Fig. 3A). These reductions in TCR/CD3 expression remained stable at days 7, 10 and 14 post-transfection, with less than 2% variability in TRAC-1 editing efficiencies for both methods (Fig. 3A). A statistically significant difference in the percentage of TCR/CD3^−^ T cells was observed between *µVS* and electroporation for all time points post-transfection.

In addition to quantifying TCR editing efficiency, we also evaluated the number of TCR knockout cells and the effects on cell viability over time for each transfection method. While we observed a statistically significant difference in TCR editing efficiency between *µVS* and electroporation, both methods yielded equivalent numbers of modified T cells by day 7, with these levels maintained at days 10 and 14 post-transfection. At 24 h post-transfection, we detected >90% viable cells in samples processed via *µVS* compared to 73% with electroporation. Peak viabilities of 98% and 85% were observed at days 7 and 10 with *µVS* and electroporation, respectively. Unlike electroporation, cells processed via *µVS* had viabilities comparable to non-transfection control samples for the duration of the experiment (Fig. 3B). In addition, we also observed a consistent enhancement in cell viability with *µVS*, with a 9 to 16% increase in the percentage of live cells observed with *µVS c*ompared to electroporation at each time point post-transfection (Fig. 3C). Furthermore, we quantified the number of live T cells after transfection and observed a 3.4-fold increase in live T cells achieved with *µVS* (8 × 10^8^ cells) compared to electroporation (2.3 × 10^8^ cells) at day 14 post-transfection. Similar to the non-transfection controls, *µVS*-processed samples experienced a 160-fold expansion in the number of live T cells at day 14 compared to the numbers observed at 24-h post-transfection. Unlike *µVS*, electroporation led to a substantial reduction in the proliferation rates of live T cells observed after transfection, with a 46-fold expansion in live T cells quantified at day 14 and proliferation curves that did not track with corresponding non-transfection controls (Fig. 3D).

**Figure 4:**
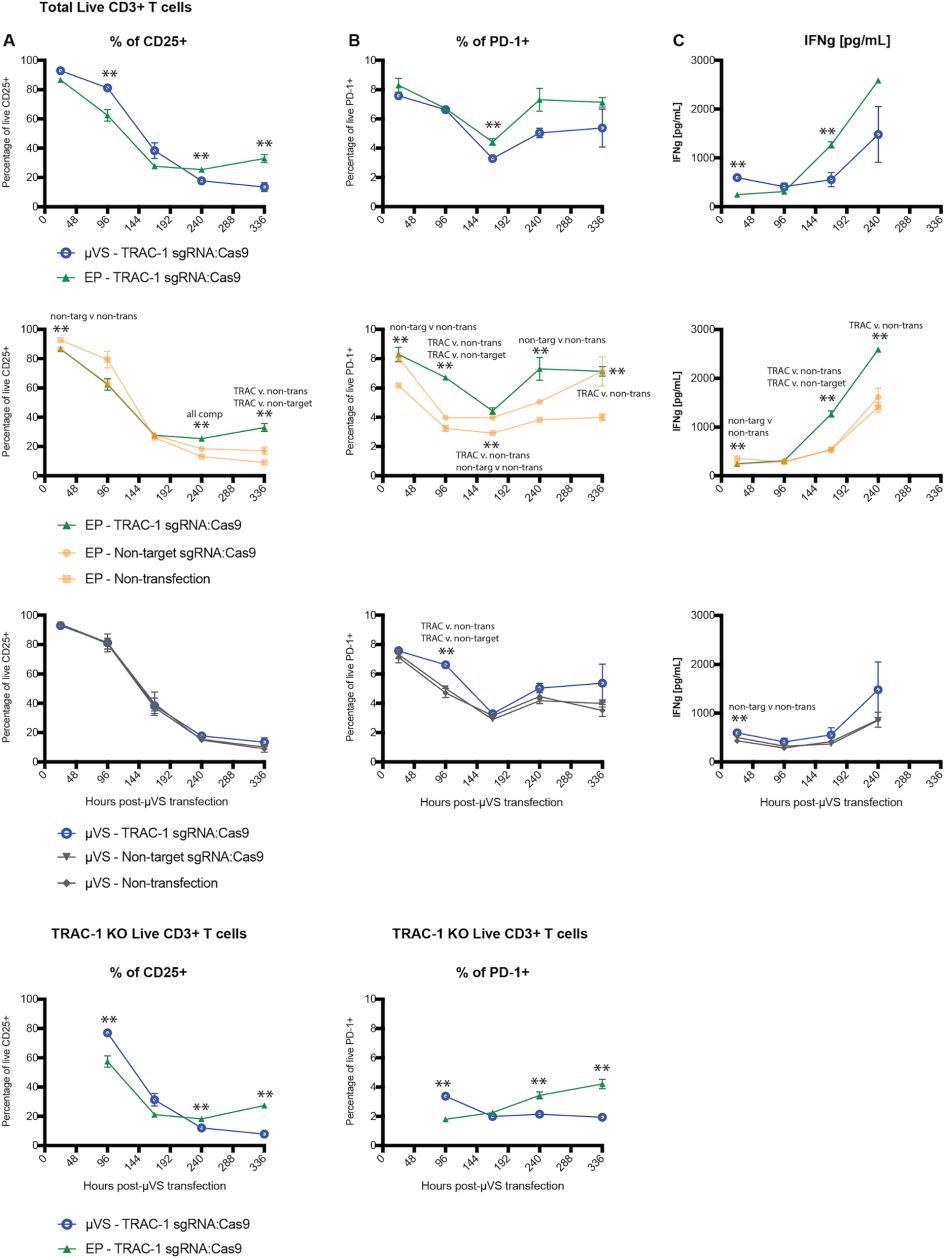
Surface marker expression and cytokine secretion levels minimally perturbed with Cas9 and *µVS*. (**A**) CD25, and (**B**) PD-1 expression levels were quantified in cells transfected with *µVS* or electroporation. Surface marker expression levels were measured in the total live cell and TRAC-1 KO live population via flow cytometry at Days 1, 4, 7, 10 and 14 post-transfection. (**C**) ELISA quantification of IFNγ supernatant levels in transfected and non-transfected cells at Day 1, 4, 7, and 10 post-transfection. Non-targeting gRNA as a Cas9–RNP complex served as a non-editing control. Cells mixed with P3 buffer (EP—non-transfection) or Opti-MEM buffer (*µVS*—non-transfection) that were not transfected served as non-transfection controls for electroporation and *µVS* samples, respectively. Data represent mean ± SD of n = 3 samples per condition. *P < 0.05, **P < 0.01 by unpaired, two-tailed, heteroscedastic *t* tests. Figure generated using Adobe Illustrator Creative Cloud (adobe.com/products/illustrator.html) and GraphPad Prism 9 (graphpad.com/scientific-software/prism/).

Electroporation has been shown to augment the expression of functional T cell surface markers and cytokines associated with their diminished function in vitro^8^. Therefore, to better understand the effect of transfection methods on T cell phenotypes, we quantified the expression of CD25 and PD-1 in cells transfected via *µVS* or electroporation compared to non-transfection controls. CD25 is an important component of the IL-2 receptor and early T cell activation marker as well as memory phenotype mediator^9^. Statistically significant differences in CD25 expression levels were observed between electroporated samples and non-transfection controls on day 1, 10 and 14 post-transfection. Specifically, we observed a consistent downregulation of CD25 expression ranging from 5 to 10% in live T cells processed via electroporation compared to non-transfection controls in early time points. Conversely, a statistically significant elevation in the levels of CD25 expression from 5 to 15% of live T cells was observed in electroporated samples compared to non-transfection controls at days 10 and 14 post-transfection. A similar trend was also observed in TRAC-1 KO live cell populations. However, unlike electroporation, no significant difference in CD25 expression was detected in cells transfected with TRAC-1 or non-targeting RNPs via *µVS* compared to non-transfection control cells at any time point, indicating that the expression of CD25 is less affected by *µVS* than it is by electroporation (Fig. 4A). For clarity, cells mixed with P3 buffer (Fig. 4, EP—non-transfection) or OptiMEM buffer (Fig. 4, *µVS*—non-transfection) that were not transfected served as non-transfection controls for electroporation and *µVS* samples, respectively.

Similar to CD25, the expression of PD-1, a T cell marker of exhaustion during early activation^10^, is also significantly affected by electroporation, with elevated levels observed at days 7 and 10 post-transfection compared to *µVS*. Similar to total live T cells, a statistically significant increase in PD-1 expression was also observed at the same time points in TRAC-1 KO cells generated vial electroporation compared to *µVS*. Furthermore, increased levels of PD-1 expression were observed in cells transfected with TRAC-1 or non-targeting RNPs via electroporation compared to non-transfection controls at days 4, 7, and 14 post-transfection. In contrast, PD-1 expression levels in live T cells were minimally perturbed when transfected via *µVS*, with PD-1 expression comparable to levels detected in T cells of non-transfection control samples (Fig. 4B). These results highlight that the process of electroporation augments PD-1 expression and potentially promotes exhaustion of T cells, while *µVS* preserves expression levels most similarly to the unperturbed native T cell state.

In addition to surface marker expression, we also quantified the supernatant levels of pro-inflammatory cytokine, interferon-gamma (IFNγ), for 14 days following *µVS* and electroporation. Like PD-1 expression, we observed a statistically significant increase in the concentration of IFNγ present at 7 days post-transfection with electroporation compared to *µVS*. A similar trend was also observed in our comparison of electroporated versus non-transfection samples, where a statistically significant increase in the levels of IFNγ were detected at days 7 and 10. By day 14 post-transfection, IFNγ levels were 2.3-fold higher in electroporated samples compared to *µVS*-processed samples when delivering TRAC-1 targeting RNP, and 1.5-fold higher when delivering non-targeting RNP (Fig. 4C). These elevations in IFNγ production in the absence of TCR engagement highlight an additional change in T cell phenotype that could alter cell function as a result of electroporation^11^, with a preservation of the normal levels of IFNγ maintained with *µVS*.

### Efficient genome editing with Cas9 and e*µVS*

**Figure 5:**
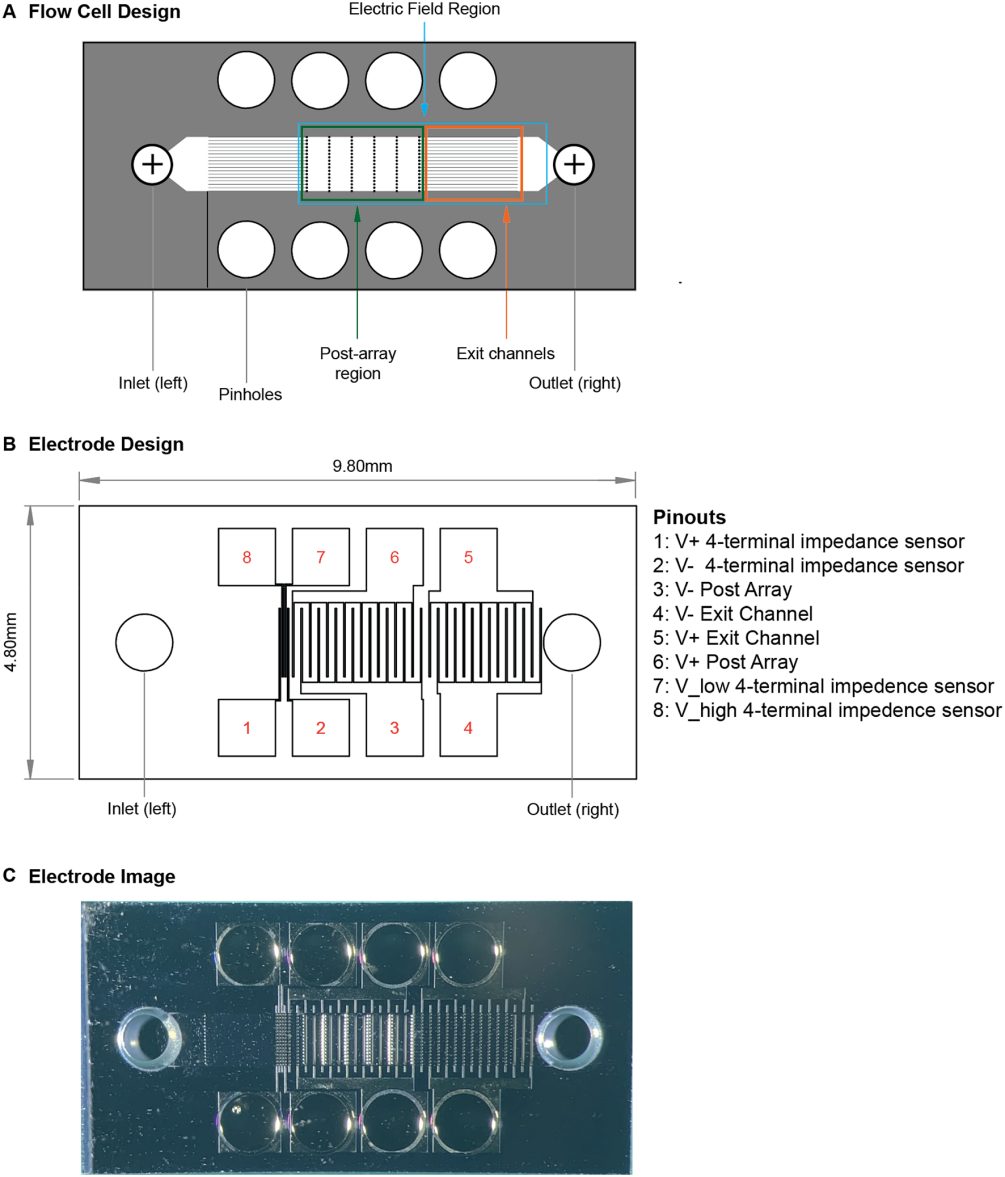
e*µVS* device and electrode designs. (**A**) 4.8 × 9.8 mm deep reactive ion etched silicon substrate contained a previously reported^6^ 40 µm deep flow cell and containing an array of 40 µm diameter posts without splitter plates (see Supplemental Figure 1B) and 1 mm pinholes for electrical access. (**B**) Each flow cell substrate is anodically bonded to a laser-machined borofloat lid. The laser-machined lid contained an electrode array for (1) measuring the electric field with a 4-terminal impedance sensor, (2) an interdigitated electrode array over the post array and (3) an interdigitated electrode array over the exit channels. Both interdigitated electrode arrays contained 25 µm wide electrodes spaced 125 µm apart. When (2) and (3) were operated at the same time with a 30 V DC offset, this enabled an up to 2.4 kV cm^−1^ applied electric field to pulse the cells 29 times over a total distance of 4.35 mm. (**C**) Optical micrograph of a 4.8 × 9.8 mm anodically-bonded *eµVS* device taken from the optically transparent borofloat lid side of the device containing the 800 µm laser machined fluidic inlet and outlet. Figure generated using Adobe Illustrator Creative Cloud (adobe.com/products/illustrator.html).

**Figure 6:**
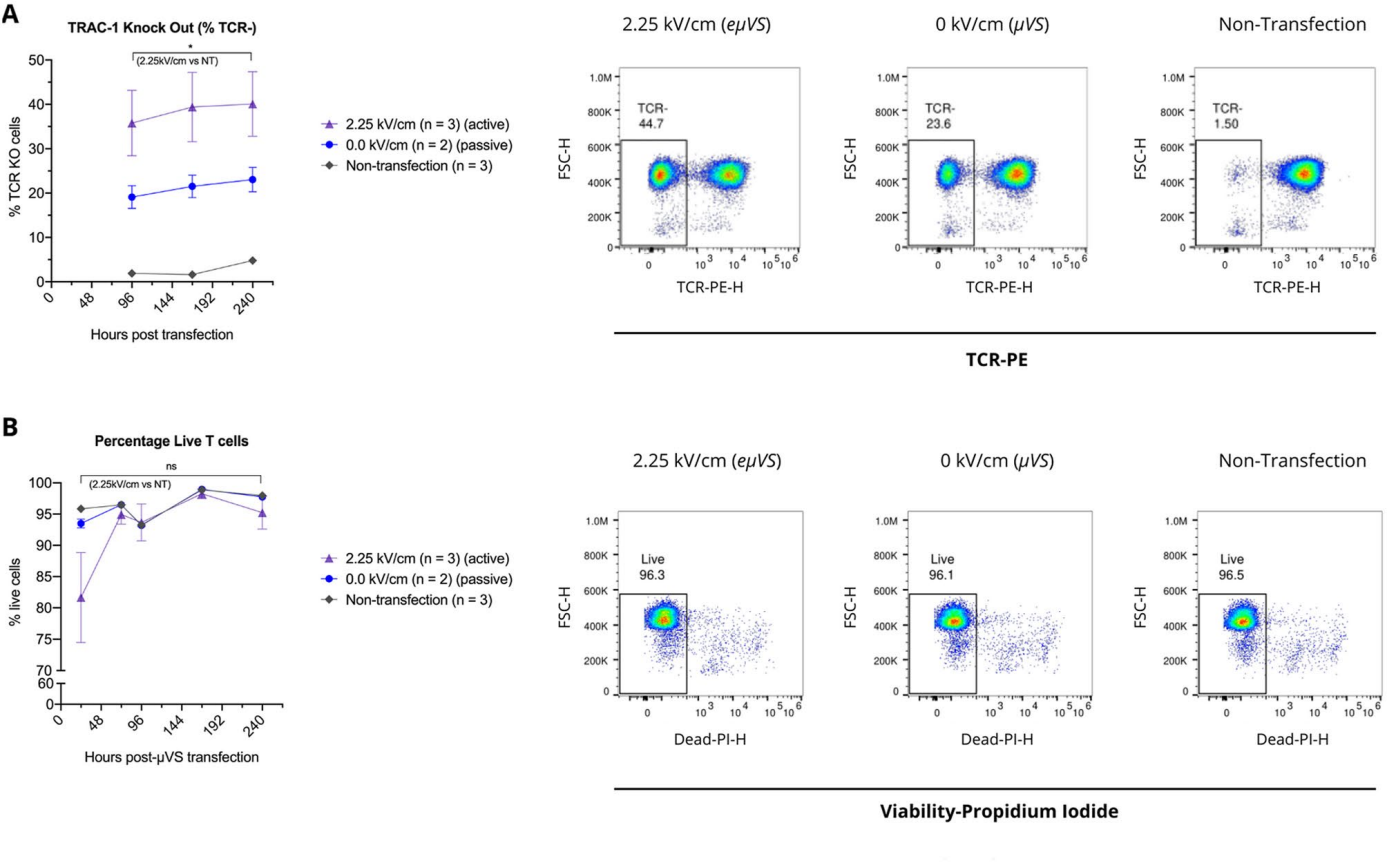
Application of electric field with *µVS* (e*µVS*) promotes intracellular delivery and TRAC-1 knockout. TRAC-1-targeting Cas9–RNPs were delivered to activated primary human CD3^+^ T cells via e*µVS* with an applied electrical field. Quantification of (**A**) percentages of TCR knockout cells and (**B**) viable T cells were quantified via flow cytometry at multiple timepoints post-transfection. TCR knockout levels were measured via TCRα/β staining and quantified as a percentage of TCRα/β-negative cells and the total number of live cells. e*µVS* with no applied electric field (0 kV cm^−1^) and non-transfection samples served as controls. Data represent the mean ± SD of n ≥ 2 samples per condition and ≥ 2 independent experiments. Statistical analysis performed between groups indicated on graphs. Dot plots representative of replicate samples. P < 0.05 by unpaired, two-tailed, heteroscedastic *t* tests. Figure generated using Adobe Illustrator Creative Cloud (adobe.com/products/illustrator.html) and GraphPad Prism 9 (graphpad.com/scientific-software/prism/).

Our experiments with *µVS* resulted in an equivalent yield of Cas9-edited T cells compared to electroporation. Therefore, we hypothesized that incremental enhancements in knockout efficiencies with the maintenance of high cell viabilities could result in an increased number of edited T cells surpassing electroporation. Thus, we sought to increase Cas9 RNP uptake in T cells via integration of interdigitated electrodes in our microfluidic devices to apply an electric field alongside *µVS*, called e*µVS* (Fig. 5)^12,13^. Preliminary experiments with OptiMEM as a processing media indicated a preferred applied electric field strength of 2.25 kV cm^−1^ where significant bubbling and clogging was observed above 2.25 kV cm^−1^ (data not shown). e*µVS* resulted in a 1.8-fold increase in editing efficiency (38.4%) relative to *µVS* (21.4%) while maintaining greater than 80% cell viability 24 h after processing (Fig. 6A). Interestingly, there was no statistically-significant difference in cell viabilities for *µVS*, *eµVS* and the non-transfection control within 72 h after processing with greater than 90% viable cells observed through day 10 for all conditions (Fig. 6B).

## Discussion

### Splitter plate simulations and experimentation

The use of splitter plates of variable lengths allowed us to analyze the influence of vortex shedding on delivery efficiency and cell viability in a manner where the influence of pressure changes and vorticity experienced by cells could be reasonably decoupled. Splitter plate designs were able to completely attenuate vortex shedding with a splitter ratio less than 0.5 with only partial levels of vortex shedding occurring in devices with a splitter ratio of 0.25. The stepwise reduction in cell viability observed at the 0.5 splitter ratio indicates single-phase simulation may not account for the onset of vortex shedding with particle-laden flows or those containing suspended cells. The highest levels of vortex shedding was observed in a previously reported *µVS* device design with no splitter plate^6^. All of this is in agreement with the fundamental splitter plate studies performed by Unal and Rockwell at similar object Reynolds numbers^7^.

Interestingly, a trend between these simulation parameters (i.e. total spanwise hydrodynamics fluctuation force and the PNWI) and the eGFP expression efficiency of mRNA and T cell viability is observed. As the splitter ratio of the *µVS* device decreases, the delivery efficiency increases, which is correlated to the rise of the total spanwise hydrodynamics fluctuation force and vortex shedding frequency in simulations. Similarly, previous studies have also demonstrated a negative correlation between delivery efficiency and cell viability^6^. In the *µVS* devices containing splitter plates, a comparable trend emerged with enhanced delivery efficiency correlating with a reduction in splitter plate lengths and reduced 24 h cell viability.

Discontinuities were also observed in simulation and empirical results highlighted in our analysis of the *µVS* device with a 0.5 splitter ratio. Despite simulating an absence of vortex shedding and minimal spanwise fluctuating hydrodynamic forces, eGFP expression was observed in the 0.5 splitter plate ratio devices. We speculated that the delivery of eGFP mRNA in cells could be due to the presence of early-onset, cell-induced vortex shedding that was not captured in our single-phase simulations in addition to the pressure changes caused by constricted flow between posts. Single-phase simulations are limited in their ability to capture nuanced vortex shedding in the presence of suspended cells and will likely require transient, multi-phase approaches to simulate suspensioned cells to better understand the contribution of vortex shedding on delivery efficiency. Multi-phase simulations with particle-laden flows are currently under development and further development of high-speed imaging at or above the Nyquist sampling frequency is required to verify those simulations.

### Genome editing with Cas9 and *µVS*

The Cas9 genome editing system has emerged as a promising approach to generate cell-based immunotherapies without the use of viral vectors along with an array of other practical applications^2,3,14^. Recently, T cells engineered to express an exogenous T cell receptor targeting tumor antigens via delivery of Cas9–RNP and DNA templates have been shown to mount effective anti-tumor responses in vitro, in murine models^15,16^, and in early stage clinical trials^5^. To explore the utility of *µVS* as an intracellular delivery method for Cas9 genome editing, we delivered Cas9 protein complexed to sgRNA targeting the T cell receptor alpha locus (TRAC-1) to knock out the expression of the endogenous T cell receptor and permanently modify activated primary human T cells. To demonstrate the feasibility of *µVS* as a tool for activated T cell genome editing, we performed a head-to-head comparison of *µVS* with an established electroporation protocol^15^. With *µVS,* we demonstrated ~ 25% editing efficiency in live T cells at 4 days post-*µVS*, compared to 95% observed in electroporated samples. Both transfection techniques resulted in stable genome editing which persisted for an additional 10 days for the duration of the experiment. Cell viability was also minimally impacted with *µVS*, with > 90% viable cells achieved at 24 h and maintained for all time points thereafter, exceeding the product release criteria for the clinical use of current CAR-T cell therapies^4^. Unlike *µVS*, a substantial negative impact on cell viability was observed as a result of electroporation, with ~75% viable cells observed at 24 h post-transfection and 9 to 16% lower viability observed at all time points compared to *µVS* and control samples. When comparing delivery methods and the variation in editing efficiencies, the contribution of double stranded breaks (DSBs) and the subsequent DNA repair process to cell viability, expansion and functional activity should be considered. The lower viability observed in the samples electroporated with non-targeting sgRNA indicates that electroporation alone, and not the process of DSBs and DNA repair, has a negative impact on cell viability. These results support electroporation as the primary contributor to adversely impact cell viability and expansion based on the minimal difference in the percentage and proliferation of live T cells for cells electroplated with TRAC-1-sgRNA:Cas9 and non-target:Cas9 control. These significant and permanent reductions in cell viability highlighted in our study are hallmarks of electroporation, challenging its adoption as a practical intracellular delivery method for clinical and commercial-scale cell therapy manufacturing^8,17^.

Accompanying delivery efficiency and viability as important factors to generate effective cell therapies, the ability to synthesize a sufficient number of modified cells to dose patients is a critical factor in successful treatment in the appropriate time frame^4,5^. Because of this, the expansion rate of genome edited T cells following transfection is of critical importance. While we observed a nearly fourfold increase in TRAC-1 editing with electroporation compared to *µVS*, the superior rate of expansion in *µVS* samples produced an equivalent number of genome edited T cells by 7 days after transfection. In addition, a 1.8-fold increase in TRAC-1 editing efficiency was achieved with the inclusion of an electric field coupled with *µVS*, *eµVS*, indicating that an enhancement in genome editing can be achieved with minimal impact to cell viability. Interestingly, TRAC-1 editing efficiencies demonstrated with e*µVS* were comparable to those reported in the first-in-human phase I clinical trial with multiplex Cas9 editing^5^. These results highlight that *µVS* and *eµVS* can likely be leveraged to minimize the time required to generate sufficient numbers of edited T cells for clinical applications beyond electroporation, without compromising cell quality. This is particularly important in light of a recent in vivo study demonstrating that a reduction in cell culture time (i.e. 3 days) enhanced anti-leukemic activity in CAR-T cells at a 6-fold lower dose^4^. The rapid gene editing time frames coupled with the development of *µVS* design with higher cell processing capacity, highlights the potential of *µVS* and *eµVS* to substantially reduce manufacturing times required to generate T cell immunotherapies, like CAR-T and TCR-T therapies at clinical and commercial scales.

### Enhanced genome editing with Cas9 and e*µVS*

The challenge of intracellular delivery stems from the impermeability of the plasma membrane. Electroporation seeks to overcome this challenge by creating temporary pores in the cell membrane through the application of a short, high voltage electric pulse (i.e. 100 µs, 5 kV cm^−1^)^18^. As the phospholipid bilayer is charged by the electric field, the transmembrane potential will reach a certain threshold where pores begin to form on the cell surface^19^. The size and duration of these transient pores are largely dependent on the strength and pulse width of the electric field. Studies show that the larger the pore is the more rapidly it will close once the electric field is removed^19^. To take advantage of this brief window of large-molecule permeability, electroporation (via Lonza’s Nucleofection) uses a second, longer pulse with an assumed lower electric field strength (4 A cm^−2^ and 40 ms) to augment the uptake of extracellular cargo^18^. This second longer pulse provides electrophoretic force which acts on the charged cargo in suspension to drive it through the transient pores and into the cytoplasm. *µVS* uses hydrodynamic forces over an estimated 200 µs period, rather than a 100 µs, 5 kV cm^−1^ electrical pulse, to permeabilize the plasma membrane. The temporary pores are formed, however, *µVS* must rely on diffusion to move cargo into the cell. Here, we demonstrate the utility of *eµVS*, the combination of the hydrodynamic conditions of *µVS* along with a specifically tailored electrical field, in delivering Cas9–RNP to activated primary human T cells. Assuming an average cell speed of 10 m s^−1^, the *eµVS* device in this study would result in an estimated total electric field exposure time of 435 µs or 644-fold less than with continuous flow microfluidic electroporation^20^. Relative to electroporation, *eµVS* replaces the 100 µs, 5 kV cm^−1^ electric field with gentle hydrodynamic membrane poration step^6^ and enhances uptake with an electrophoretic field over a 92-fold reduced total electric field exposure time^18^. Cumulatively, this enables *eµVS* to efficiently deliver Cas9 genome editing constructs to T cells while also minimizing impacts on cell viability relative to various forms of electroporation.

Importantly and during preliminary studies, it was determined that commercial electroporation buffers (such as those used by the Lonza Nucleofector and BTX Electroporation system) are extremely harsh on T cells after exposure times exceeding 30 min. For *µVS* and *eµVS*, however, we relied on commercially-available OptiMEM that does not significantly impact T cells viabilities with longer incubations and is available as a cGMP-grade reagent. This may further enable *µVS* and *eµVS* as a practical and gentle intracellular delivery platform. Additional work is required to determine the influence of different processing media and their respective conductivities on *eµVS* performance.

Another important consideration during *eµVS* is the electric field. When using this *eµVS* device and the above protocol, cells are pulsed with 29 electric fields about 125 µm in length with a 25 µm gap between fields due to the electrode array geometries (see Fig. 5B,C). This is similar to exposing cells suspended in OptiMEM to a 2.25 kV cm^−1^ applied electric field at 66.7 kHz with an 83.3% duty cycle for 29 pulse widths over 435 µs. In practice, the use of a 4-terminal impedance sensor (Fig. 5B) could be used to measure the electric field strength. Varied flow speeds and cell trajectories as a result of vortex shedding in the post array region are likely to create a varied electric field exposure and this may result in the greater error bars seen with *eµVS* relative to *µVS* (Fig. 6A). Reducing cell-to-cell processing variability for *eµVS* could be achieved by determining the minimum flow cell width that allows for efficient *µVS.* This would be similar to the above splitter plate study, but with reduced flow cell widths rather than splitter plates increasing in length. This would ensure less variability in cell trajectories due to recirculation resulting in more uniform electric field exposure. This minimal flow cell width could then be used for applications requiring low numbers of genome edited cells in discovery-stage workflows like high throughput screening and this same flow cell geometry could then be arrayed across a substrate for applications where significant numbers of genome edited cells are required like cell therapy^5^.

## Conclusion

Our investigation of microfluidic vortex shedding via simulations and experimentation provides a better understanding of the fluid dynamics contributing to *µVS* as a method for intracellular delivery. We demonstrated a novel application of *µVS* for genome editing activated human T cells with the Cas9 genome-editing system. Compared to electroporation, *µVS* resulted in superior cell viability and proliferation, and preservation in cell surface marker expression and cytokine secretion levels relative to non-transfection cell controls. Despite discrepancies in editing efficiency, we observed an equivalent number of genome edited T cells with *µVS* and electroporation. Further, we demonstrated enhanced Cas9-based gene editing through e*µVS*, the application of *µVS* coupled to a brief electric field, with a 1.8-fold increase in TCR knockout editing efficiency with low impact on cell viability.

Studies investigating transgene insertion using Cas9–RNPs with different DNA templates and targets^15,16^, transposon/ase system^21^ with e*µVS,* and the influence of *µVS* on functional T cell proteins, including CD25^9^, in cancer immunotherapy and autoimmune diseases, are currently ongoing. Future efforts to further delineate the mechanistic underpinnings of *µVS* will focus on direct comparisons of multi-phase, particle-laden CFD simulations with high-speed imaging and will be used to better study the effects of fluctuating lift force and PWNI on intracellular delivery. Ultimately, these results will expedite the development of *µVS* and *eµVS* devices optimized to deliver a variety of constructs and cell types and in indications like cancer immunotherapy^22^ and autoimmune disorders^23,24^.

## Methods

### Splitter plate device design and simulation

A total of five splitter plate device CAD geometries were designed and constructed using OnShape software. The geometries were computationally meshed and simulated with OpenFOAM software to evaluate and investigate the influence of vortex shedding on cell viability, mRNA delivery and subsequent eGFP expression. Splitter ratios for these devices were 1.0, 0.75, 0.5, 0.25 and 0 while separation ratios were 0, 0.75, 2, 3.25 and 10, respectively.

Our CFD computational domain consisted of structured (hexagonal) mesh with a total of 30 million grid points. Mesh independent studies were performed to ensure the numerical flow results were sufficiently resolved without the influence of mesh resolution. The finest resolutions used in the device geometry, located near the cylindrical posts, were at a 2 µm. This resolution was considered sufficiently to resolve flow shear layers with a total of four grid points across the device in the spanwise direction. The coarsest resolutions, located near the inlet and outlet channels of the device, were set at 8 µm. Slip boundary conditions were not used on walls except for the inlet and outlet channels, which had pressures of 134.7 and 14.7 PSIA, respectively, to create a 120 PSIG pressure drop in each device. A single-phase laminar Reynolds number was used with Opti-MEM at 23°C (⍴ = 1011.4 kg m^−3^, *μ *= 9.586 × 10^4^ kg m^−1^ s^−1^) as processing fluid. 3D transient simulations were performed with OpenFOAM 5.0’s transient PimpleFOAM single phase solver. A total of 3.5 ms flow through time was simulated with a time-step of 1 µs. An initial transient time of 1.5 ms was necessary to allow flow to become fully developed with the numerical schemes. Therefore, a total of 2.0 ms of statistical numerical data was collected to resolve flow structures behaviours with a flow spectral frequency from 5 to 500 kHz. Numerical solutions required 19,800 to 22,800 CPU-hours over 4 days with 198 parallel cores on Rescale and Amazon Web Services supercomputers. Rescale’s C4 Instant cluster type was used with Intel Xeon 2.9 Hz CPUs with 3.8 GB of memory per core.

Vortex shedding fluctuations were quantified based on hydrodynamics fluctuating forces acting on cylindrical posts. Vortex structure scales were visualized qualitatively with the aid of spanwise vorticity fluctuation contours and Q-criterion iso-surfaces. To quantify the hydrodynamics performance of each splitter plate device, a performance indicator referred to as the Post Near Wake Indicator (PNWI) was created (SI—Detailed Simulation Analysis). PNWI values were calculated for each splitter plate device and a ratio was created relative to a baseline configuration (splitter plate ratio = 1.0) to rank their hydrodynamic performance.

### *µVS* device fabrication

Devices were fabricated using previously reported, industry standard semiconductor techniques^6^. Briefly, *µVS* device and device features (posts, channel thickness, inlet and outlet channels) were manufactured by (1) generated a digital rendering of the microfluidic using Onshape CAD software, (2) preparing a wafer substrate and (3) performing a series of metallisation, inspection, photolithography, liftoff, and laser drilling on the substrate.

### eGFP mRNA and *µVS* splitter plate study

Primary human CD3^+^ T cells were isolated from healthy donor peripheral blood mononuclear cells (PBMCs) via immunomagnetic negative selection (STEMCELL Technologies). PBMCs were ethically sourced using Informed Consent Forms and protocols approved by the WCG Institutional Review Board (IRB). The WCG IRB (which includes the Western IRB, Copernicus Group IRB, Aspire IRB, New England IRB, and Midlands IRB) is the name of the Institutional Review Board that has approved the study protocols and Informed Consent Forms for all these collection sites and lots. Please refer to the WCG IRB website for information, including their Statement of Compliance and details of the name change in 2020. Informed Consent was obtained from all subjects and all subjects were over the age of 18 for the lots collected. Donations were performed in the United States in compliance with applicable federal, state, and local laws, regulations, and guidance. Donors were pre-screened for general health and viral status, including HIV-1, HIV-2, Hepatitis B, and Hepatitis C.

For thaw and culture, cryopreserved T cells were expanded using anti-CD3/CD28 dynabead T cell activator (ThermoFisher) at a 1:3 bead:cell ratio for 2 days, followed by debeading and *µVS*.

All solutions processed through the device and device were filtered prior to use with a 0.22 μm filter to remove particulates. For device processing, T cells were debeaded, washed, resuspended in Opti-MEM (Thermofisher, 31985062) and filtered with a sterile 40 µm cell strainer. eGFP-encoding mRNA (TriLink) was delivered at 200 µg mL^−1^ (50 µg mRNA) into activated T cells at 1.5 × 10^7^ cells mL^−1^ (3.75 × 10^6^ cells) in a total volume of 250 µL with a driving pressure of 120 PSIA. Sample rig and tubing were sterilized before use via 70% ethanol wipe down and flush. Immediately prior to *µVS*, samples were mixed thoroughly by gentle pipetting, mounted in the sample reservoirs and driven pneumatically though the device for intracellular delivery by *µVS*. Processed samples were collected in warmed (37 °C) media (XVIV020 + 5% human serum, Lonza), and cultured at 1.0 × 10^6^ cells mL^−1^ supplemented with IL-2 at 100 IU mL^−1^ (Peprotech). Each splitter plate condition was evaluated in triplicate. eGFP and cell viability levels were quantified by propidium iodide viability staining (ThermoFisher) and flow cytometry (Attune, ThermoFisher Scientific) at 24 h post-*µVS*.

### Genome editing with Cas9, *µVS* and electroporation

Cas9 (Invitrogen) and TRAC-1-specific single-guide RNAs (sgRNAs, Synthego), as a Cas9–RNP complex (1:1 Cas9:gRNA molar ratio) were delivered primary human CD3^+^ T cells after 24 h following 2 days of Anti-CD3/-CD28 Dynabead (ThermoFisher) stimulation at a 1:3 bead:cell ratio. Dynabeads were removed by placing cells on a cell separation magnet for 2 to 5 min. Immediately before *µVS*, de-beaded cells were centrifuged for 10 min at 1500 rpm, aspirated, resuspended in Opti-MEM and filtered with a 40 µm cell strainer. Cas9–RNPs were delivered at 5.0 × 10^–5^ pmol RNP cell^−1^ to 5.0 × 10^6^ activated T cells sample^−1^ at 5.0 × 10^7^ cells mL^−1^ in a total volume of 100 μL, with an applied driving pressure of 120 PSIA. For electroporation, de-beaded cells were aspirated and resuspended in 100 µL of Lonza electroporation buffer P3. 5.0 × 10^6^ activated T cells were electroporated per cuvette using a Lonza 4D X-unit electroporation system with pulse code EH115. Processed samples were collected in warmed (37 °C) media (XVIV020 + 5% human serum, Lonza), and cultured at 1.0 × 10^6^ cells mL^−1^ supplemented with IL-2 at 100 IU mL^−1^ (Peprotech). IL-2 at 100 IU mL^−1^ was supplemented every 2 to 3 days after transfection. Each condition was evaluated in triplicate. Levels of TCR, CD3, PD-1 and CD25 expression and cell viability and cell expansion were quantified by live/dead staining and the following antibodies via flow cytometry: anti-TCRα/β-PE (306708, BioLegend), anti-CD3-AF700 (11-0039-42, ThermoFisher), Sytox Blue Viability Stain (S34857, ThermoFisher), anti-PD-1-APC (ThermoFisher, S34857), anti-CD25-SuperBright 702 (ThermoFisher, 67-0259-42). Flow cytometry analysis was performed on days 1, 4, 7, 10 and 14 post-transfection and FlowJo software (BD). Cell culture supernatant levels of IFNγ were quantified using Human IFNγ Quantikine ELISA Kit and manufacturer’s recommendations (DIF50, R&D Systems).

### Flow cytometry

Flow cytometric analysis was performed on an Attune NxT Acoustic Focusing Cytometer (ThermoFisher). Surface staining was performed by pelleting cells and resuspending in flow buffer (1% human serum in PBS) with antibodies for 30 min at 4°C in the dark. Cells were washed twice in flow buffer prior to resuspension and analysis.

### *eµVS* device fabrication and operation

*eµVS* devices were designed, fabricated and operated in a similar manner without a splitter plate (Fig. 5). The addition of an electric field was achieved by adding platinum interdigitated electrodes to the lid of a *µVS* flow cell design with industry standard lithography methods^6^. Due to the additional fabrication requirements, the flow cell was etched in silicon then anodically bonded to a borofloat lid (see Fig. 5). Fluidic access to the flow cell was achieved by laser drilling 800 µm diameter through holes in the borofloat lid and thru silicon vias enabled electrical access. 100 nm thick and 25 µm wide platinum electrodes were spaced 125 µm apart to achieve a ratio of the electrode spacing to the flow cell height suitable to create uniform electric fields between the interdigitated electrodes^13^. The electric field was applied using a standard 120 V DC power supply (Nice-Power, R-SPS1203D) coupled with a customized harness to distribute the current evenly across the platinum electrodes. In addition, we used an oscilloscope (Tektronix, TBS 1052B) connected to the leads from the DC power supply to monitor the applied electric field strength. Using this circuit, we applied up to 30 V of continuous DC voltage to the electrodes with a gap space of 125 µm resulting in an maximum applied electric field strength of 2.4 kV cm^−1^.

## Supplementary Information

Supplementary Information.
