# Supplementary material for: Numerical optimization of microfluidic vortex shedding for genome editing T cells with Cas9
Source: Sci Rep. 2021 Jun 3;11:11818. doi: 10.1038/s41598-021-91307-y (PMC8175688; doi:10.1038/s41598-021-91307-y)
Supplement: Supplementary file 1 — Supplementary Information. [file 41598_2021_91307_MOESM1_ESM.docx]

**Supplemental Information**

**Numerical optimization of microfluidic vortex shedding for genome editing T cells with Cas9**

Justin A. Jarrell^1^, Brandon J. Sytsma^1^, Leah H. Wilson^1^, Fong L. Pan^1^, Katherine H.W.J. Lau^1^, Giles T. S. Kirby^2^, Adrian A. Lievano^1^ and Ryan S. Pawell^1^

1. Indee Labs, Berkeley, CA, United States

2. Future Industries Institute, University of South Australia, Mawson Lakes, SA, Australia

**Supplemental Figures**

*Jarrell JA et al.* Numerical optimization of microfluidic vortex shedding for genome editing T cells with Cas9

**
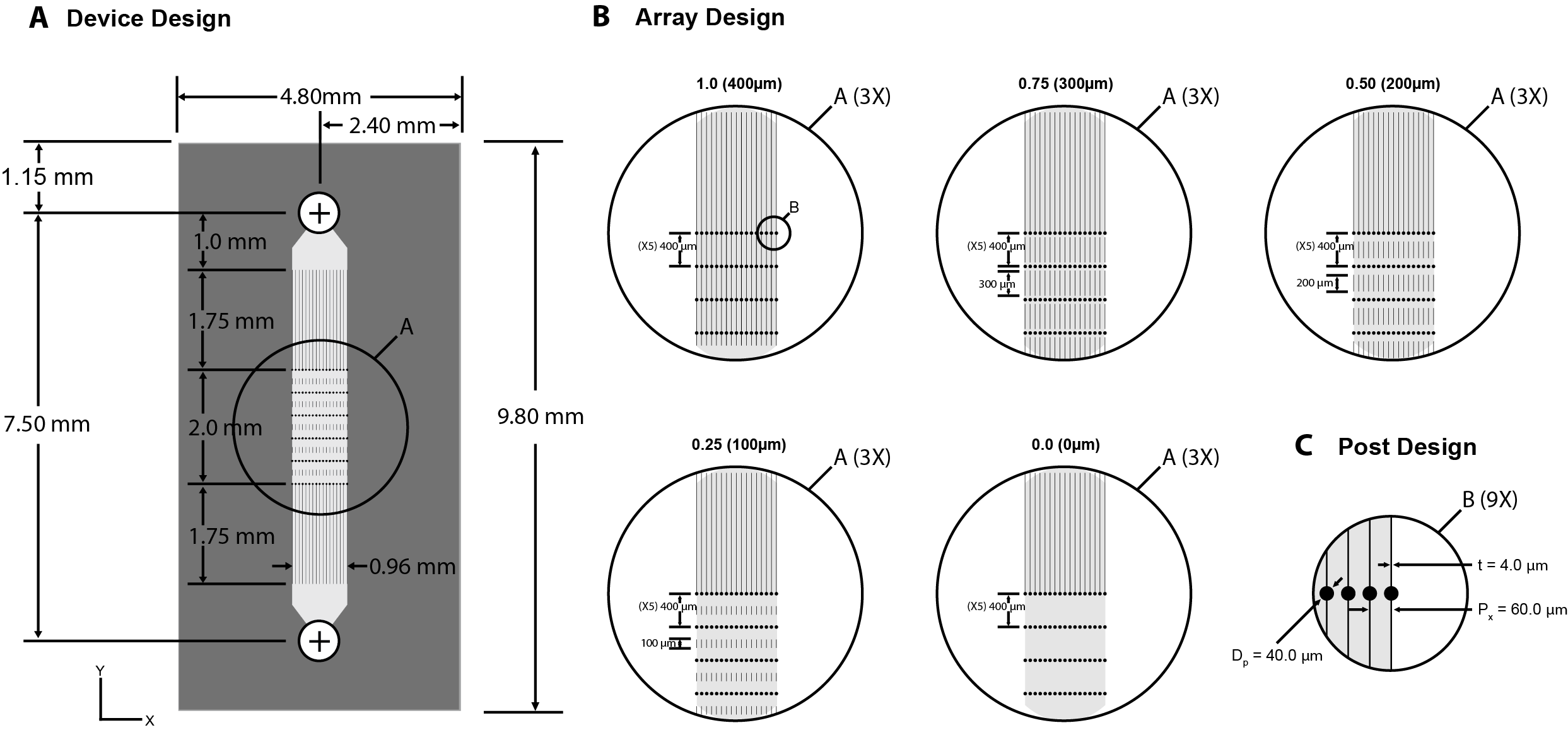
**

**Supplemental Figure 1. *µVS* device, array and post designs.** **A**, *µVS* devices were fabricated with a 4.8 x 9.8 mm footprint with a 7.5 mm long and 40 µm deep previously-reported flow cell design^6^. Flow cells contained inlet and outlet channels flanking 6 columns of posts of 17 posts per column representing the post array region. **B**, Array designs for five *µVS* devices with varied lengths splitter plates (400-100 µm) or no splitter plate (0.0, 0 µm). **C**, All posts were identical with 40 µm diameter and depth. Splitter plate walls were of 4 µm width creating 60 µm wide channels. Figure generated using Adobe Illustrator Creative Cloud ([adobe.com/products/illustrator.html](http://adobe.com/products/illustrator.html)).


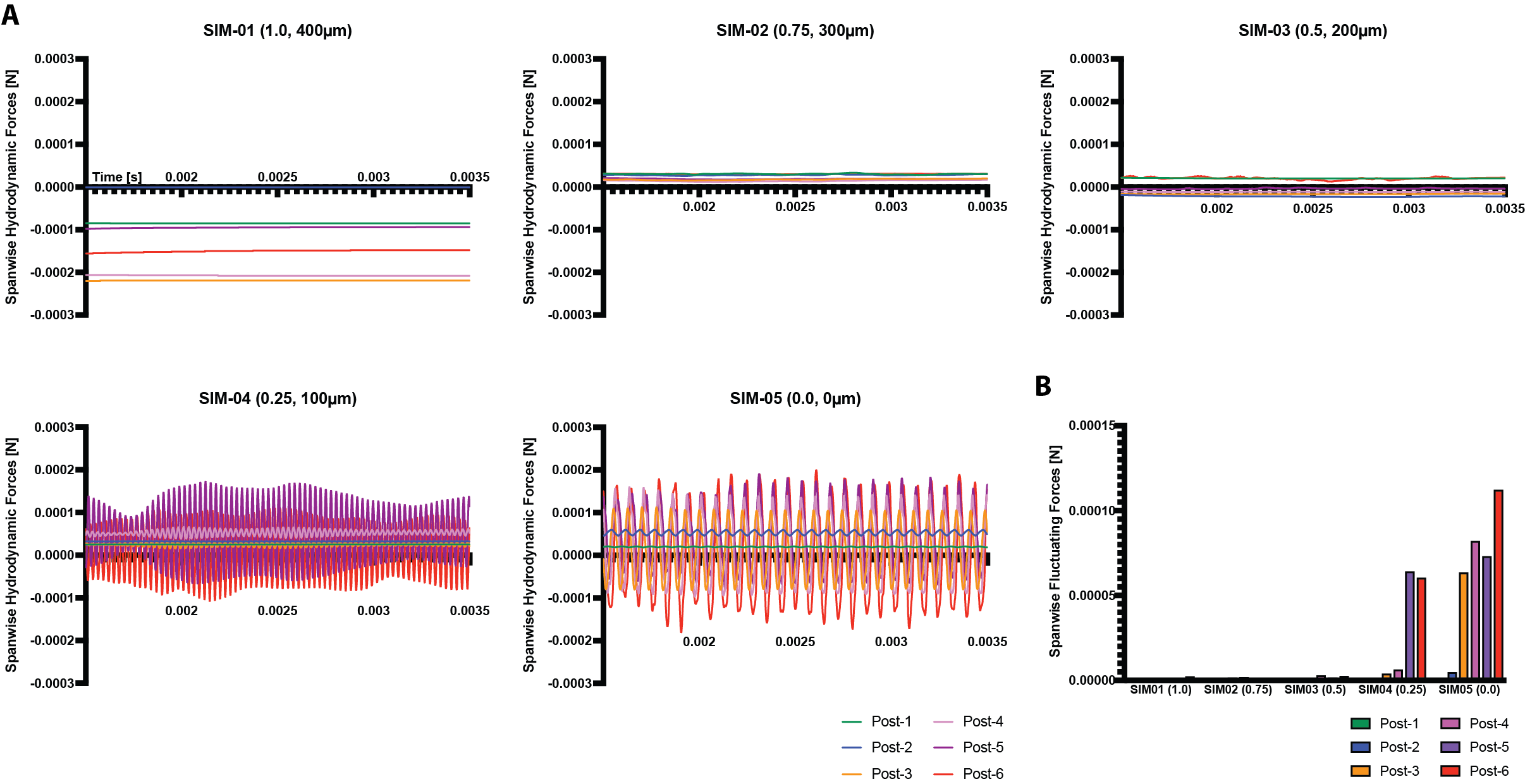


**Supplemental Figure 2. Transient force analysis of *µVS* splitter devices.** (A) Spanwise hydrodynamics forces [N] on each post column of *µVS* devices with 0.0 - 1.0 splitter plate ratios as a function of time. (B) Spanwise hydrodynamic fluctuations on each post column (1 - 6) for each splitter device design. Figure generated using Adobe Illustrator Creative Cloud ([adobe.com/products/illustrator.html](http://adobe.com/products/illustrator.html)), GraphPad Prism 9 ([graphpad.com/scientific-software/prism/](https://www.graphpad.com/scientific-software/prism/)) and OpenFOAM 5.0 ([openfoam.org/version/5-0/](https://openfoam.org/version/5-0/)).

**
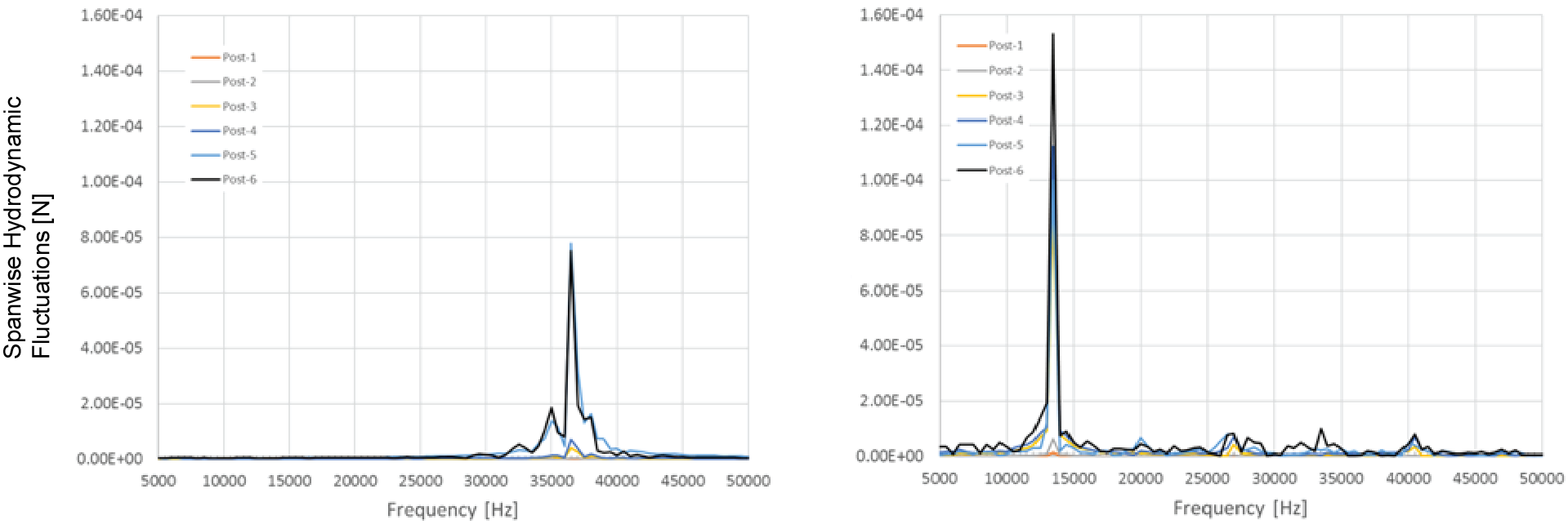
**

**Supplemental Figure 3.** Simulated Spanwise Hydrodynamic Forces Spectral Frequency of *µVS* devices. Spanwise hydrodynamics forces spectral amplitude of both SIM04 (left, 0.25 splitter ratio) and SIM05 (right, 0.0 spitter ratio). Figure generated using Adobe Illustrator Creative Cloud ([adobe.com/products/illustrator.html](http://adobe.com/products/illustrator.html)), GraphPad Prism 9 ([graphpad.com/scientific-software/prism/](https://www.graphpad.com/scientific-software/prism/)) and OpenFOAM 5.0 ([openfoam.org/version/5-0/](https://openfoam.org/version/5-0/)).

**
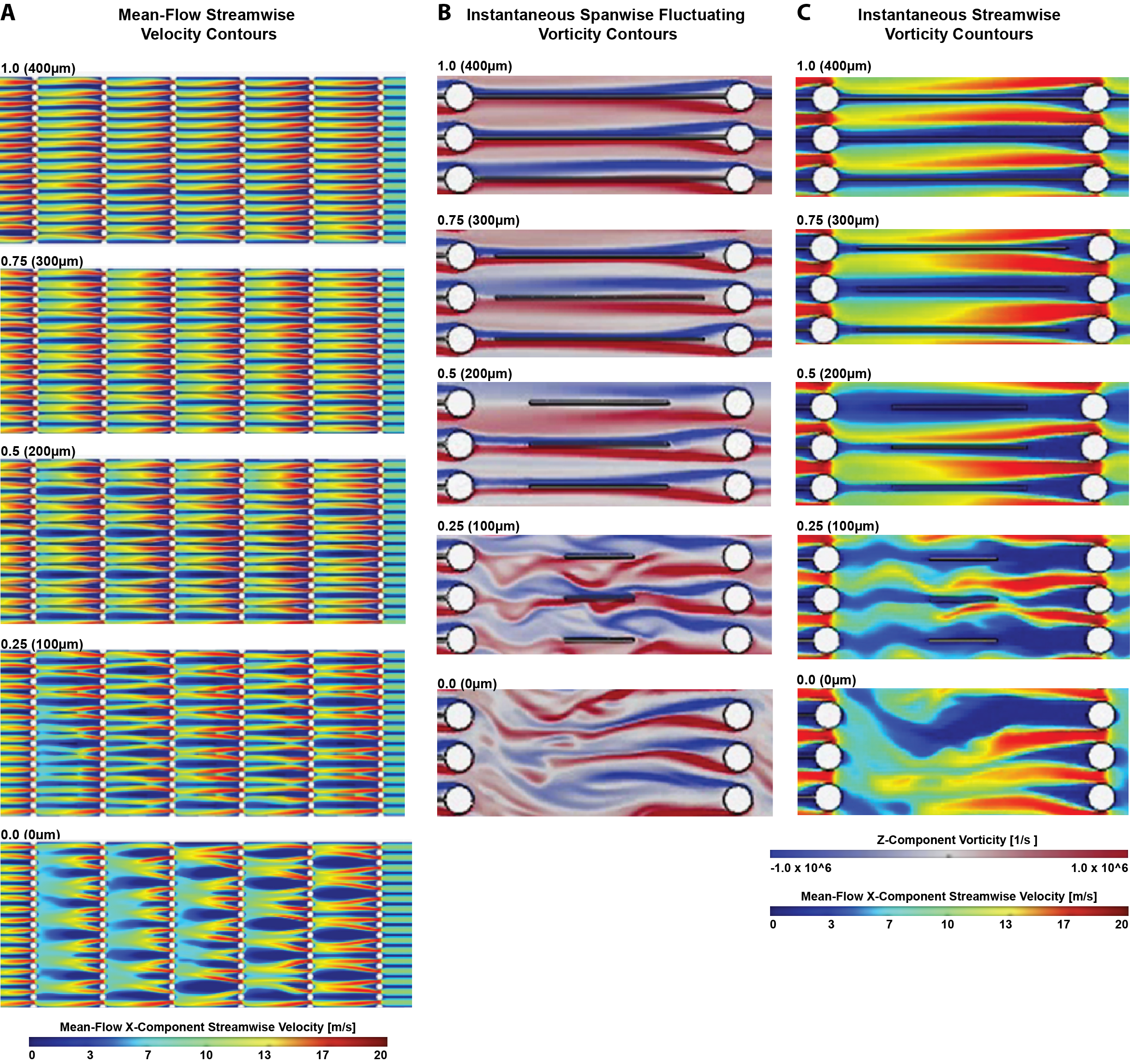
**

**Supplemental Figure 4.** Simulated Flow-Field Contours as Mean-Flow Streamwise, Instantaneous Spanwise Fluctuating and Instantaneous Streamwise Velocity Contours of *µVS* devices. Mean-flow streamwise **A**, and instantaneous spanwise **B**, and instantaneous streamwise vorticity **C**, contour distribution for all *µVS* device designs. Figure generated using Adobe Illustrator Creative Cloud ([adobe.com/products/illustrator.html](http://adobe.com/products/illustrator.html)) and OpenFOAM 5.0 ([openfoam.org/version/5-0/](https://openfoam.org/version/5-0/)).


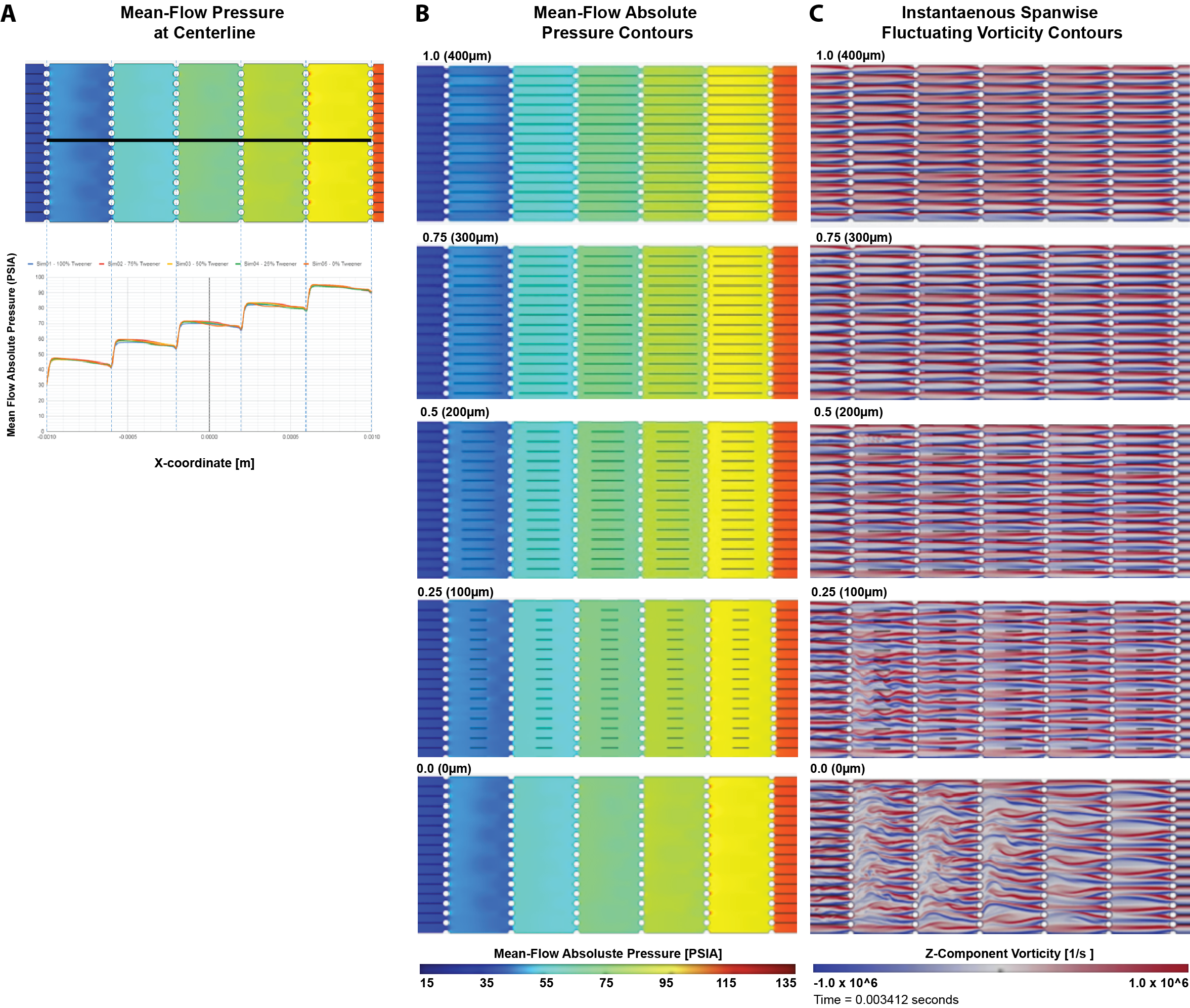


**Supplemental Figure 5.** Simulated Mean-Flow Hydrodynamic Pressure Distributions and Contours and Instantaneous Spanwise Fluctuating Vorticity Contour of *µVS* devices. **A**, Mean-flow hydrodynamics pressure distribution at the centerline of the *µVS* device. Distribution is from the 1st to 6th (of 6) post-columns. Hydrodynamic pressure uniformly distributed in spanwise direction (top). Local pressure drops were detected across each post column in the streamwise direction for all design configurations (bottom). **B**, Absolute pressure and **C**, instantaneous spanwise fluctuating vorticity magnitudes in the flow fields pseudo-colored for all *µVS* devices designs. Figure generated using Adobe Illustrator Creative Cloud ([adobe.com/products/illustrator.html](http://adobe.com/products/illustrator.html)) and OpenFOAM 5.0 ([openfoam.org/version/5-0/](https://openfoam.org/version/5-0/)).

**
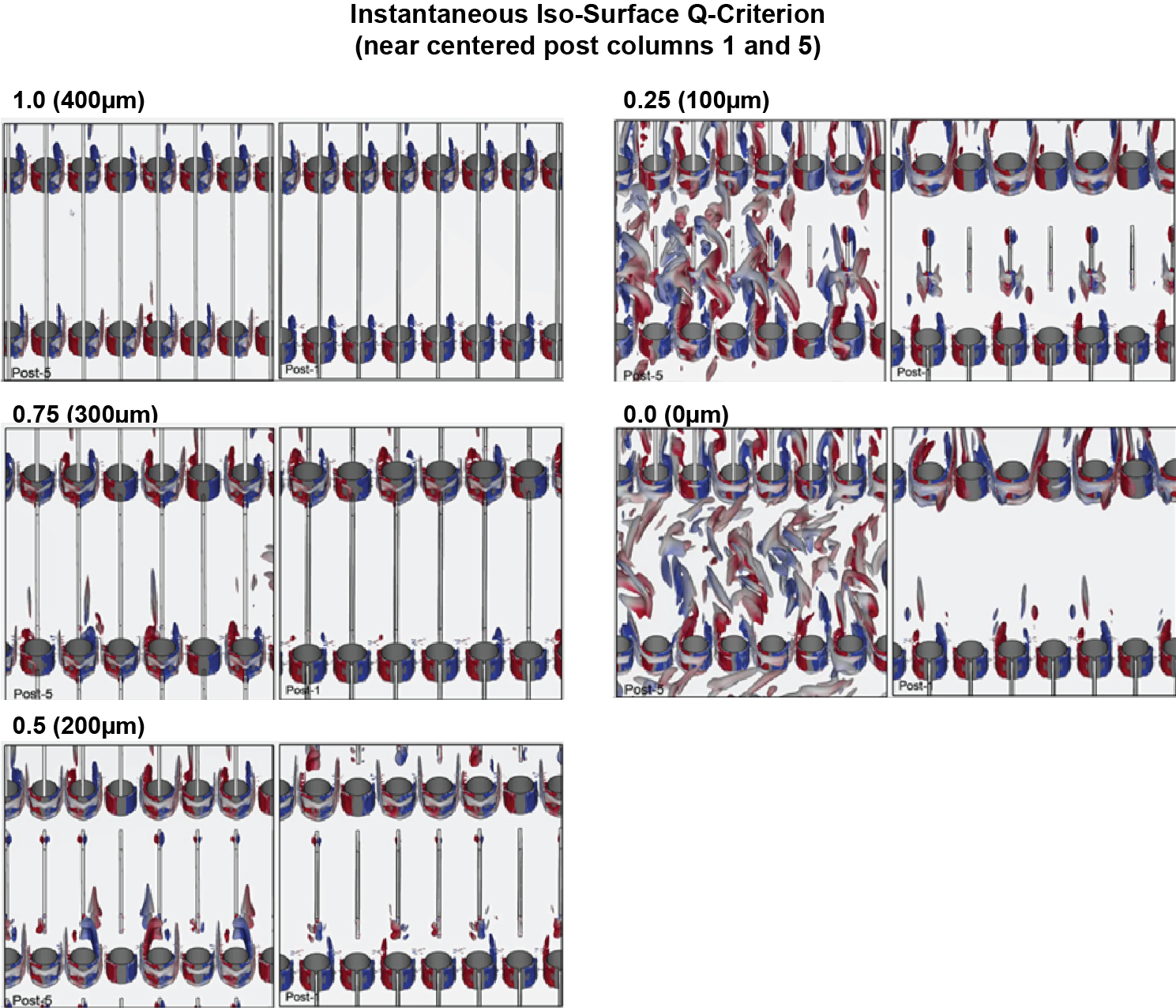
**

**Supplemental Figure 6.** Simulated Q-criterion Iso-surfaces to visualize vortex structures in *µVS* devices. Q-criterion in flow-field simulated to visualize structures in three-dimensional space. Simulation images show Q-criterion iso-surface at post-columns 1 (right) and 5 (left) for all *µVS* device designs. Figure generated using Adobe Illustrator Creative Cloud ([adobe.com/products/illustrator.html](http://adobe.com/products/illustrator.html)) and OpenFOAM 5.0 ([openfoam.org/version/5-0/](https://openfoam.org/version/5-0/)).

**Supplements**

*Jarrell JA et al.* Numerical optimization of microfluidic vortex shedding for genome editing T cells with Cas9

**Supplement 1. Detailed Simulation Analysis**

**Centerline Pressure Distribution** Supplemental Figure 1 shows the mean-flow hydrodynamics pressure distribution at the centerline of the device. The distribution depicted the pressure variations at the first to last column. The presence of the splitter plates had minimal impact on the hydrodynamics pressure distribution. Local pressure drops were consistently identified across each post column in the streamwise direction for all device designs. Generally speaking, such local pressure drops varied at ~20 psig (1.4 ATM) at by-pass flow duration of 2 µs for all device designs. The local pressure drop was largely dictated by the local flow restriction due to closely aligned posts positioned along each column.

In between columns, the hydrodynamics pressures were uniformly distributed in the spanwise direction. Interestingly, these distributions remained uniform in the absence and presence of vortex shedding. Along the centerline of the device, local pressure recovery developed in the streamwise direction extending downstream into the far-field region starting from the base of each post. Such pressure recovery development was located at the far-field wake formation as flow convected downstream.

**Mean-flow Hydrodynamics Pressure Contour and Instantaneous Spanwise Fluctuating Vorticity Contours** Supplemental Figure 1 shows flow-fields colored by absolute pressure and instantaneous spanwise fluctuating vorticity magnitude for all devices. Hydrodynamics pressure in between columns were uniformly distributed in spanwise direction for all configurations in the absence and presence of vortex shedding conditions.

As indicated in Supplemental Figure 1, fluctuating vortices were predominant in both 0.0 and 0.25 splitter ratio devices. Such spanwise fluctuations were considered the footprint of vortex shedding pairs in their oscillating modes. Red and blue colors in the contours represented counter-rotating vortices pairs with the same strength magnitude. Conversely, hydrodynamics vorticities remain undistorted (remain stationary without oscillation) in the 0.50, 0.75 and 1.0 splitter ratio devices in spanwise direction behind a majority of posts. In particular, more dominant vorticity fluctuations at the last 3 post columns were present in the 0.25 splitter ratio devices whereas uniform fluctuation behaviors across all columns was observed in the 0.5 splitter ratio device.

The hydrodynamic pressure distribution contours indicated that the fluid pressure remains uniform in regions between consecutive post columns. Such hydrodynamic pressure distribution was unperturbed in the presence of vortex shedding structures in 0.0 and 0.5 splitter plate ratio devices (Supplemental Figure 1). Pressure uniformity was self-sustained by a pair of counter-rotating vortices with similar strengths oscillating at both symmetrical sides of the post (Supplemental Figures 1 & 2). The pressure distribution remaining undisturbed in the presence of vortex shedding inferred that the flow dynamics in the device would remain unchanged. Therefore, it is speculated that the flow rates for all splitter plate ratio devices remained consistent at a given applied pressure.

Therefore, we can conclude that in the presence of the vortex shedding (i.e. 0.25 and 0.0 splitter ratio), the uniform hydrodynamics pressure distribution is undisturbed since the vortex structures are fluctuating in equal and opposite strength and direction at both symmetrical sides of the post. It is expected that such a synchronous oscillating pattern of the vortex shedding in both 0.0 and 0.25 splitter cases shall not disrupt the uniformity of the pressure distribution that is similarly found in 0.5-1.0 splitter ratio cases.

In addition, the simulation results also provided evidence of vortex structures present in the device as quantified by the vortex shedding frequency. Vortex shedding frequency was quantified from the periodic form of oscillation of the vortex structures behind the posts. It was found that only 0.25 and 0.0 splitter plate ratio devices exhibited consistent periodic oscillation of the dominant vortex shedding, at rates of 36 kHz and 13.5 kHz, respectively (Table 1, Supplemental Figure 3). Minimal to no vortex structures were observed in the 0.5, 0.75, or 1.0 splitter plate ratio configurations with zero vortex shedding frequencies detected. It should be noted that these simulations are for single-phase flow that may only be representative of the flow of suspended cells through a *µVS* device. Furthermore, vortex shedding spectral analysis also suggested that vortex suppression occured when the separation ratio was less than 3.25 (SIM01-03, splitter plate ratio 1.0 - 0.5), similar to previous reports^7^. Numerical analysis also revealed that vortex formation inside the wake region occurred as a result of two (‘pair’) of counter-rotating vortices developed behind a post (Supplemental Figure 4). In the 0.0 splitter plate ratio device, the wavelength of its vortex pairs was approximately 6 times the post diameter (240*µm*). This is consistent with the computed vortex convection speed of 3 m s^-1^ at the frequency rate of 13.5 kHz based on wavelength-frequency relationship. However, in the 0.25 splitter plate ratio device, the presence of this splitter plate (with a separation ratio of 3) interfered with oscillation downstream of the posts while full oscillating wavelengths were undetectable in the 1.0, 0.75, and 0.5 splitter plate ratio devices (Supplemental Figure 2B & 5). Devices simulated with a separation ratio of 3 (SIM04, 0.25 splitter plate ratio) resulted in a higher frequency of vortex oscillation with lower spanwise hydrodynamic forces when compared to SIM05 (0 splitter plate ratio) (Table 1, Supplemental Figure 3). Overall, the inclusion of various splitter plates allowed for the incremental attenuation of vortex shedding in a manner that enabled us to computationally determine vortex shedding frequency and total spanwise fluctuating hydrodynamics forces. Subsequently, we were able to compare these computed results to biological experiments in order to assess correlations between cell viability, mRNA delivery efficiency, vortex shedding frequency and/or total spanwise fluctuating hydrodynamics forces. Achieving a similar result empirically through high speed imaging and/or micro-particle image velocimetry is simply not feasible due to the sampling frequency and cell media composition requirements, respectively.

**Mean-flow Streamwise Velocity Contours** Supplemental Figure 2 shows the mean-flow streamwise velocity distributions for all design configurations. The mean-flow streamwise velocity contours provide insight to the extent of wake formation regions in the device. In the 0% splitter ratio case, the wake regions were widely spreaded in both far and near-field regions indicated by low negative streamwise velocity colored by blue. In contrast, both the 1.0 and 0.75 splitter ratio devices had fewer and narrower wake regions in most fluid domains compared to all other devices. In general, the number of wide wakes found in the domain increased with the reduction in splitter ratio. The presence of a splitter plate at the finite length of 0.50-1.0 splitter ratios showed an attenuation of near-field vortex shedding and therefore prohibited downstream (far-field) wake formation.

The large number of wide wake regions present in the 0.0 splitter ratio device is speculated to permit and promote the *µVS* intracellular delivery process. It is anticipated that the vortex shedding observed inside the wide wake regions are likely to prolong the flow duration (increase cell residence duration) for the intracellular delivery process to occur. Therefore, it is critical to conduct a set of studies with different column pitch separations for achieving the optimal performance. This work is currently ongoing.

Instantaneous flow parameters such as vorticity and streamwise velocity are useful to evaluate vortex behaviours in both near-field and far-field regions. Supplemental Figure 2 shows a close up view of a pair of spanwise oscillating vortex structures identified in 0.0 and 0.25 splitter ratio devices. In the 0.0 splitter ratio device, the spanwise oscillating vortex pairs are present, starting from each side of the post. Such vortex shedding oscillation behavior extended further downstream to the free wake region. Far-field wake development also occurred in devices with separation ratios >3.2. In all other cases, the near-field vortex shedding was attenuated in near-field and prohibited any further wake development in far-field.

**Q-criterion Iso-Surfaces** Q-criterion detection technique was used to visualize vortex structures in three-dimensional space. Supplemental Figure 3 demonstrates the 3D vortex structures detected in between columns 1 and 2 (right) and 5 and 6 (left). Large scale vortices were detected between column 5 and 6 for 0.25 and 0.0 splitter ratio devices. Large scale vortices were found to behave in coherence as expected. In contrast, minimal vortex structures were detected upstream between column 1 and 2. These Q-criterion results were consistent with the spanwise vorticity contours previously observed and described above. For 0.25 and 0.0 splitter ratio devices, coherent vortices oscillated at much higher amplitude at downstream columns than in upstream columns.

Vortex structure behavior was also detected by measurement of Q-criterion iso-surfaces (Supplemental Figure 6). Q-criterion analysis indicated that the vortex structures were oscillating at higher amplitude in the downstream post columns (4-6) compared to upstream columns (1-3) in the flow cells of the 0.0 and 0.25 splitter plate ratio devices. Based on this, it is speculated that the number of post columns included in a device design will impact the overall device performance (i.e. delivery efficiency) due to vortex amplitude enhancement. Furthermore, the self-sustained vortex fluctuations behavior in the downstream columns are also thought to further enhance device performance as an intracellular delivery method.

**Supplement 2. Post Near Wake Indicator (PNWI)**

PNWI is defined as a Post Near Wake Indicator derived from the hydrodynamics fluctuating forces. The PNWI % calculation is based on the following steps:

1) Time history of total spanwise absolute hydrodynamics forces for each post column is recorded.

2) The standard deviation of total spanwise absolute hydrodynamics forces for each column is computed. This result gives hydrodynamics fluctuations about the mean.

3) Ratio to 0% tweener for each case is computed based on the standard deviation computed from Step 2.

4) This ratio is named “Post Near-Wake Indicator (PNWI)”. The general formula of PNWI is: *^PNWI^*^% = 00%^ √^∑(^*^F^* ^−^*^F^* ^)^

*_i mean_*2

[^∑(^*^F^* ^−^*^F^* ^)^ *_i mean_*^2^]*_ref_*× 1

where *F* is absolute hydrodynamics forces measured at each post and is averaged (mean) *_i_ F _mean_* hydrodynamics forces measured at each post.
